# Coexpression of EpCAM, CD44 Variant Isoforms and Claudin-7 in Anaplastic Thyroid Carcinoma

**DOI:** 10.1371/journal.pone.0094487

**Published:** 2014-04-11

**Authors:** Toshihiro Okada, Teruo Nakamura, Takayuki Watanabe, Naoyoshi Onoda, Atsuko Ashida, Ryuhei Okuyama, Ken-ichi Ito

**Affiliations:** 1 Division of Breast and Endocrine Surgery, Department of Surgery, Shinshu University School of Medicine, Matsumoto, Nagano, Japan; 2 Department of Surgery, Shinshu University School of Medicine, Matsumoto, Nagano, Japan; 3 Department of Surgical Oncology, Osaka City University Graduate School of Medicine, Osaka, Japan; 4 Department of Dermatology, Shinshu University School of Medicine, Matsumoto, Nagano, Japan; University of Occupational and Environmental Health, Japan

## Abstract

**Background:**

Anaplastic thyroid cancer is considered to be one of the most aggressive human malignancies, and the mean survival time after diagnosis is approximately six months, regardless of treatments. This study aimed to examine how EpCAM and its related molecules are involved in the characteristics of anaplastic thyroid carcinoma.

**Methodology/Principal Findings:**

Two differentiated thyroid cancer cell lines (TPC-1 and FTC-133), and two anaplastic thyroid cancer cell lines (FRO, ACT-1) were analyzed for expression of CD44 standard isoform (CD44s), CD44 variant isoforms, and EpCAM, and human aldehyde dehydrogenase-1 (ALDH1) enzymatic activity using flow cytometry. CD44s expression was higher in TPC-1 and FTC-133 than in the FRO and ACT-1, whereas ALDH1 activities were higher in FRO and ACT-1 than in TPC-1 and FTC-133. An inverse correlation between CD44s expression and ALDH1 activity was observed in all thyroid cancer cell lines. As for the expressions of CD44 variant isoforms, ACT-1 showed higher and FRO showed moderate CD44v6 expressions, whereas either TPC-1 or FTC-133 showed negative CD44v6 expression. EpCAM expressions in FRO and ACT-1 were higher than those in TPC-1 and FTC-133, and EpCAM expressions inversely correlated with those of CD44s. A positive correlation was observed between EpCAM expression and ALDH1 activity in thyroid cancer cell lines. In the RT-PCR analysis, the expression levels of EpCAM, caludin-7 and ALDH1 in FRO and ATC-1 cells were significantly higher than those in TPC-1 and FTC-133 cells. In clinical specimens of thyroid cancers, nuclear expression of EpCAM and high expression of CD44v6 were detected significantly more frequently in anaplastic carcinomas.

**Conclusions/Significance:**

Our study suggests the possibility that EpCAM, together with CD44v6 and claudin-7 as well as ALDH1, may be involved in the development of the aggressive phenotype of anaplastic thyroid carcinoma. Our findings may suggest a novel therapeutic strategy for treatment of anaplastic thyroid carcinoma.

## Introduction

Thyroid cancer is the most common endocrine malignancy worldwide as well as in Japan. Thyroid carcinomas are classified into the following four representative histological types: papillary, follicular, medullary, and anaplastic carcinomas. Medullary carcinoma derives from thyroid parafollicular (neuroendocrine) C cells, whereas papillary, follicular, and anaplastic carcinomas originate from thyroid follicular cells. Papillary and follicular carcinomas together are termed as differentiated thyroid carcinomas, and they generally carry a favorable prognosis. In contrast, anaplastic thyroid cancer is considered to be one of the most virulent human malignancies and the mean survival time after diagnosis is less than one year, regardless of the treatment administered [1]–[3].

It has been widely known that most of the patients with anaplastic thyroid carcinoma have previous or concomitant differentiated thyroid carcinomas, and an “anaplastic transformation” from the differentiated carcinoma to the anaplastic carcinoma is sometimes clinically observed. However, the underlying molecular mechanisms of anaplastic transformation remain poorly understood.

The expression of thyroglobulin is reduced concurrent with anaplastic transformation [4]. We recently reported that one of the UDP-GalNAc: polypeptide N-acetylgalactosaminyl transferases (GalNAcTs), GalNAc-T3 expression is higher in the well-differentiated carcinoma, whereas it is rarely detected in anaplastic carcinoma [5]. Thus, as the altered expressions of not a few molecules were observed during anaplastic transformation, there may be some critical molecules among them that accelerate the “anaplastic transformation” [6].

Epithelial cell adhesion molecule (EpCAM) is a 40 kDa transmembrane glycoprotein and is expressed on many epithelia [7], . EpCAM has been shown to play important roles in cell adhesion, proliferation, differentiation, migration, and cell cycle regulation, and its expression is frequently increased in many malignancies [9]–[13]. Furthermore, high EpCAM expression correlates with tumor grading and the prognosis of the tumors [14]–[16]. EpCAM overexpression strongly correlates with poor overall survival and bad prognosis and distinguishes patients at high risk for recurrence in a variety of cancers [9], [17], [18]. In addition, EpCAM has been demonstrated as a cancer-initiating cell marker of several solid cancers such as breast, colorectal, and pancreatic cancers [19]–[24].

CD44 is a single transmembrane protein with a large family of at least 20 variants based on differential splicing and post-translational glycosylation [25]. The expression of CD44 is regulated by the Wnt signaling pathway via β-catenin. CD44 is thought to be involved in the signature of tumor-initiating cells from the analyses of several solid carcinomas such as colon carcinomas, head and neck carcinomas, non-small cell lung cancer, hepatocellular carcinoma, and breast cancer [22], [26]–[29].

Among the CD44 variant isoforms, variant isoform v4–v7 are thought to be carcinoma-associated variants [30], [31]. Furthermore, CD44v6 expression has been described as a valuable marker for diagnosis and prognosis of cancer [27]–[29]. However, with regard to the correlation with prognosis of cancer, a positive to an inverse correlation between CD44v6 expression and prognosis have been reported, and the same conflicting findings have been observed for expression of CD44 standard isoforms (CD44s) as well.

EpCAM has been shown to interact directly with CD44v4–v7 but not with CD44s [32] and claudin-7, which is a protein required for the formation of tight junctions [33]. Of late, Kuhn et al. reported that the complex of EpCAM, claudin-7, CD44v6, and tetraspanin, rather than the individual molecules, promotes tumor progression and facilitates metastasis formation in colorectal cancer [16]. Thus, growing evidence indicates that the role of EpCAM in cancer should be further investigated in light of its interaction with these molecules. Of late, nuclear accumulation of the intracellular domain of EpCAM and a reciprocal loss of the membranous extracellular domain of EpCAM was demonstrated to predict poor prognosis of patients with thyroid cancers and is associated with reduced overall survival [34], [35]. Thus, the involvement of EpCAM in progression of thyroid cancer from an indolent to an aggressive phenotype has been suggested.

However, to the best of our knowledge, the association of EpCAM, CD44v6 and claudin-7 have not been evaluated in anaplastic thyroid carcinoma. The purpose of this study was to evaluate the involvement of these molecules as well as aldehyde dehydrogenase 1 (ALDH1) in anaplastic thyroid carcinoma and explore the mechanisms underlying transition from indolent differentiated thyroid carcinoma to virulent anaplastic thyroid carcinoma.

## Materials and Methods

### Cell lines

Papillary thyroid cancer cell line, TPC1 and anaplastic thyroid cancer cell line, FRO were donated from Dr. Yamashita at Nagasaki University [36]. Follicular thyroid cancer cell line, FTC-133 was donated from Dr. Takeda at the Fourth Department of Internal Medicine [37], Shinshu University School of Medicine. Anaplastic thyroid cancer cell line, ACT-1 was established by Dr. Ohata at Tokushima University and was kindly donated [38].

### Clinical specimens

This study was conducted according to the ethical guidelines of the Declaration of Helsinki, and specific approval was obtained from the Ethics Committee of Shinshu University School of Medicine. The patients gave their written informed consent for providing their specimens for the study, and the Ethics Committee approved this consent procedure. The specimens studied were obtained from 75 patients with thyroid cancers who were diagnosed and treated in Shinshu University Hospital from 1995 to 2005.

### Flow cytometry and cell sorting

Flow cytometric analyses and sorting were performed on single-cell suspensions derived from each cell line. ALDH1 enzymatic activity was detected using the ALDEFLUOR assay kit (StemCell Tehnologies, Durham, NC). The basis for this assay is that uncharged ALDH substrate (BODIPY-aminoacetaldehyde [BAAA]) is taken up by living cells via passive diffusion. Once inside the cell, BAAA is converted into negatively charged BODIPY-aminoacetate (BAA) by intracellular ALDH. The negatively charged BAA is then retained inside the cell, causing the cell to become highly fluorescent. Diethylaminobenzaldehyde (DEAB) was used to inhibit the ALDEFLUOR reagent, providing a negative control. Antibodies (FITC-conjugated anti-CD44s, PE-conjugated anti-CD24, and anti-EpCAM were purchased from Miltenyi Biotec (Bergisch Gladbach, Germany). PE-conjugated-CD44s, -CD44v3, and -CD44v6 were purchased from R&D Systems (Minneapolis, MN). Fluorescence activated cell analyses and sorting were done by FACS Caliber and FACS Aria III (BD Bioscience, San Jose, CA). Hoechst 33342 was used for specifically staining the nuclei of the cells.

### RT-PCR

TaqMan Gene Expression Assays for EpCAM (cat # Hs00901885_m1), ALDH1A1 (Hs00946916_m1), and β-actin (Hs99999903_m1) were purchased from Applied Biosystems (Carlsbad, CA) and mRNA levels were quantified in triplicate using Applied Biosystems 7300 Real-Time PCR system (Carlsbad, CA).

### Immunohistochemical staining and evaluation

A formalin-fixed, paraffin-embedded, 3-µm section was obtained from clinical specimens. Sections were deparaffinized in xylene, hydrated through a graded series of ethanol, and immersed in 3% hydrogen peroxide in 100% methanol for 30 min to inhibit endogenous peroxidase activity. For immunohistochemical analyses, slides were heated for antigen retrieval in 10 mmol/L of sodium citrate (pH 6.0). Sections were subsequently exposed to specific antibodies for CD44s (2C5; R&D Systems, Minneapolis, MN), CD44v6 (VFF-18; abcam, Cambridge, England), EpCAM (E144; abcam), or isotype-matched controls at appropriate dilutions. Then, sections were incubated with Histofine Simple Stain MAX PO (MULTI) (NICHIREI BIOSCIENCES, Tokyo, Japan). Stainings were revealed using Diaminobenzidine (NICHIREI BIOSCIENCES) and counterstained with aqueous hematoxylin. The intensity of staining was classified into four levels (0, 1+, 2+, 3+) for CD44s and CD44v6.

### Statistical analysis

The levels of mRNA expression obtained by real time RT-PCR were examined by Student t-test with a *P*-value of less than 0.05 were considered statistically significant. The chi-square test was used for immunohistochemical analysis of the clinical specimens.

## Results

### Basic characteristics of the thyroid cancer cell lines used in this study

We evaluated the presence or absence of *BRAF* gene mutation in the four thyroid cancer cell lines used in the study (Table 1). One anaplastic cancer cell line, FRO, had a V600E mutation in *BRAF* and the three other cell lines had the wild type *BRAF*.

**Table 1:** Basic characteristics of thyroid cancer cell lines.

Cell line	Origin	*BRAF*
TPC-1	Papillary	w/t
FTC-133	Follicular	w/t
FRO	Anaplastic	V600E
ACT-1	Anaplastic	w/t

### Expression levels of EpCAM and standard and variant isoforms of CD44 in thyroid cancer cell lines

**Figure 1:**
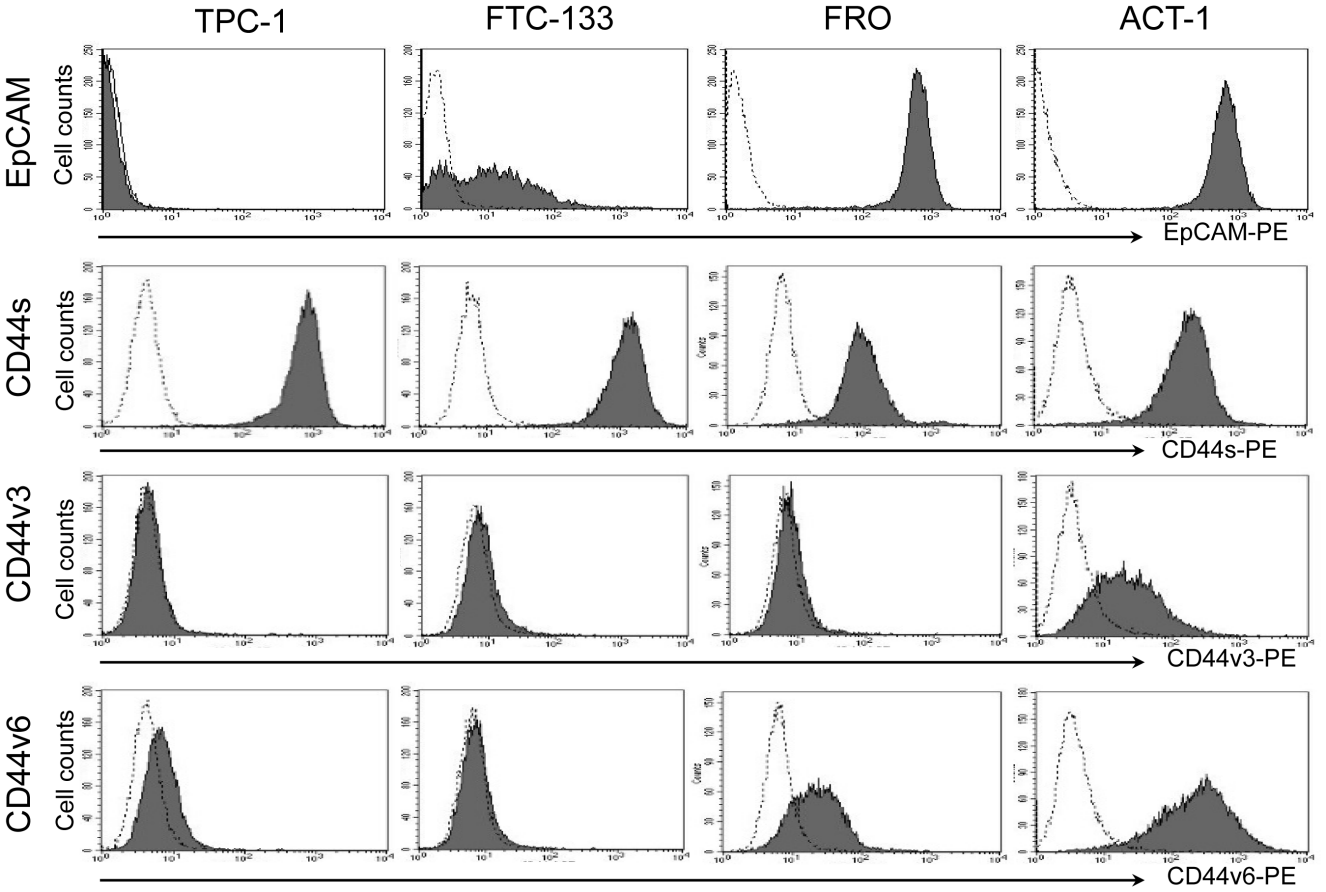
Expression of EpCAM, CD44s, CD44v3 and CD44v6 in thyroid cancer cell lines. Thyroid cancer cell lines, TPC-1, FTC-133, FRO and ACT-1 were tested by flow cytometry for expression of EpCAM, CD44s, CD44v3 and v6. Filled histograms represent positive staining for the proteins of interest, open histograms show negative control with matched isotype antibody.

The expression of EpCAM and CD44s, and two CD44 variant isoforms, CD44v3 and CD44v6, were examined by flow cytometry (Figure 1). The expression of EpCAM in FRO and ACT-1 were remarkably higher than those in the TPC-1 and FTC-133 although low expression of EpCAM was observed in FTC-133 cells.

In contrast, the expression of CD44s was higher in TPC-1 and FTC-133 cells than in FRO and ACT-1 cells. Thus, EpCAM and CD44s were inversely expressed in these four cell lines, with the anaplastic thyroid cancer cell lines expressing higher levels of EpCAM and lower levels of CD44s compared with the differentiated cancer cell lines.

With regard to variant isoforms of CD44, the expression of CD44v3 was undetectable in TPC-1, FTC-133, and FRO cells. ACT-1 cells alone showed very low expression of CD44v3. In contrast, moderate to high expression levels of CD44v6 were detected in FRO and ACT-1 cells, although the expression level was higher in ACT-1 cells. Marginal expression of CD44v6 was detected in TPC-1 cells. Thus, the anaplastic thyroid cancer cell lines expressed variant isoforms of CD44 in contrast to the reduced CD44s expression.

### Expression of EpCAM and CD44s in thyroid cancer cell lines

**Figure 2:**
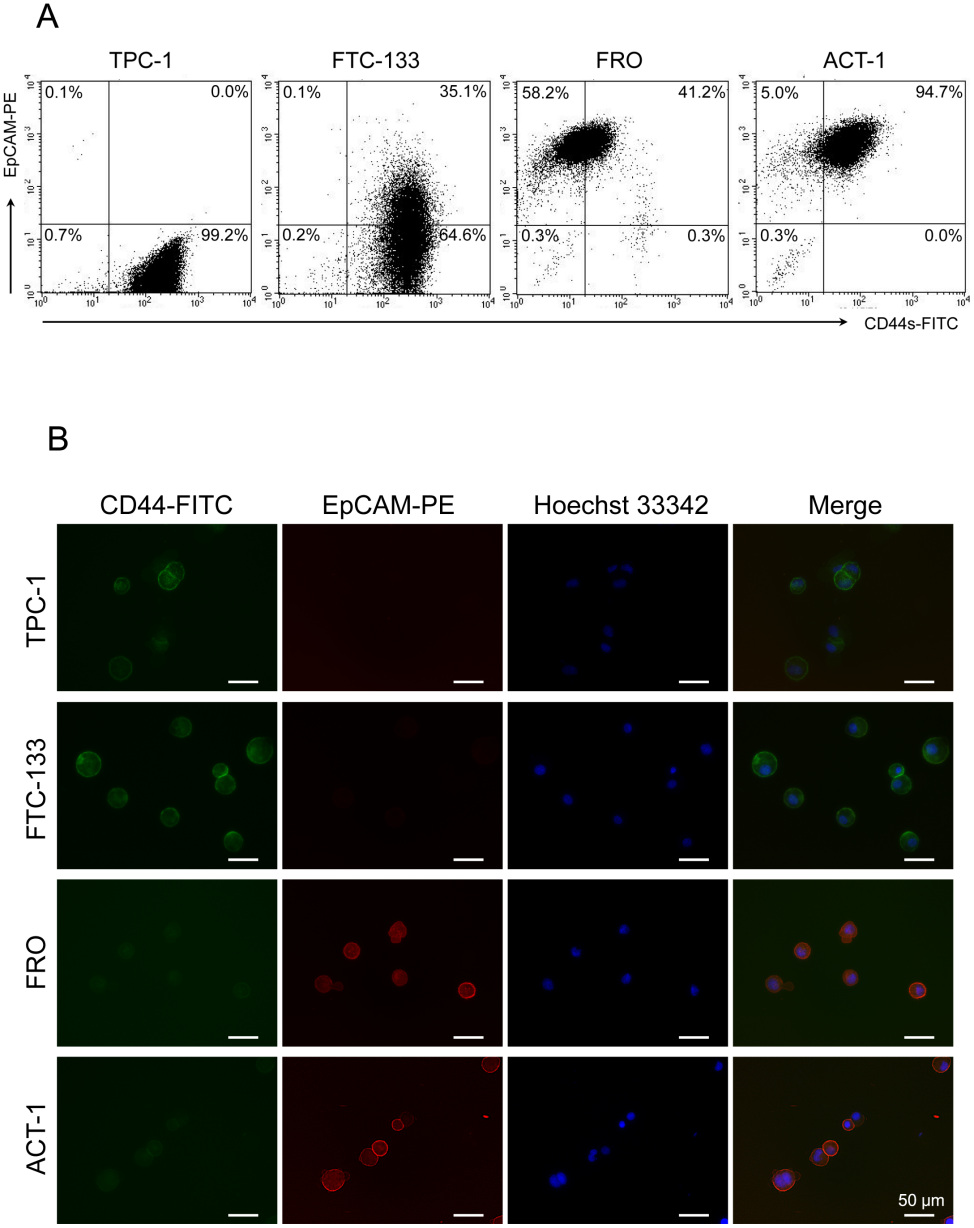
Expression of EpCAM and CD44s in thyroid cancer cell lines. (A) Four thyroid cancer cell lines were tested for expression of EpCAM and CD44s by flow cytometry. The number indicated the percentage of the cells in each subset. (B) Immunofluorescence findings of CD44s and EpCAM in indicated cell lines. The cells were directly labeled using FITC-conjugated CD44s (green) and PE-conjugated EpCAM (red) antibodies and were examined under a fluorescence microscope. Nuclei were stained blue with Hoechst 33342. Scale bar = 50 µm.

In the flow cytometry analyses, the expressions of EpCAM in FRO and ACT-1 were higher than those in the TPC1 and FTC-133 and the CD44s expression levels were lower in FRO and ACT-1. Double immunofluorescent staining of EpCAM and CD44s was conducted to detect the cells expressing these molecules in the four thyroid cancer cell lines. In TPC-1 and FTC-133, a high level of CD44s expression was detected in a majority of the cells in the flow cytometry analyses. On the contrary, a low level of expression of EpCAM was observed in these cells (Figure 2A). As for FRO and ACT-1 cells, a high level of EpCAM together with a low level of CD44s was observed in a majority of cells in the flow cytometry analyses. In the microscopic analyses, the expression of CD44s was detected at plasma membrane in most of the TPC-1 and FTC-133 cells. In contrast, EpCAM was not detected in most of the TPC-1 and FTC-133 cells, and only a fraction of cells in FTC-133 cells expressing low level of CD44s co-expressed EpCAM (Figure 2B). In the anaplastic cancer cell lines, most of the cells showed lower levels of CD44s, but higher expression of EpCAM than the two differentiated thyroid cancer cell lines. In the anaplastic cancer cell lines, EpCAM was strongly expressed at the plasma membrane, and a weak nuclear expression was detected in the cells as well. An inverse association was observed between the expression of CD44s and EpCAM in the thyroid cancer cell lines examined in this study. Moreover, the obvious difference between the differentiated cancer cell lines and the anaplastic cancer cell lines was observed with regard to the expression of these molecules.

### mRNA and protein expression of EpCAM and claudin-7 in thyroid cancer cell lines

**Figure 3:**
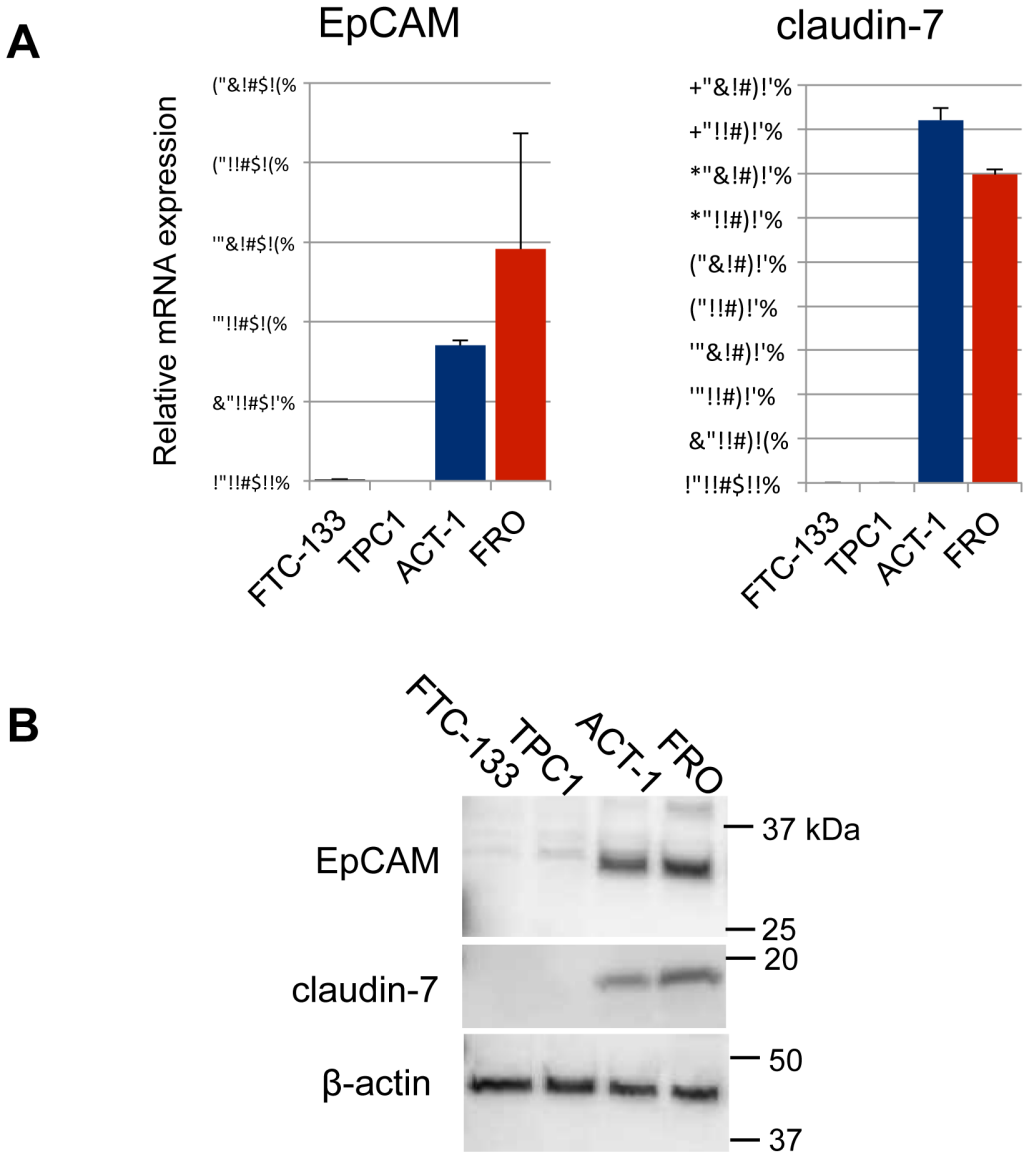
mRNA and protein expression of EpCAM and claudin-7 in thyroid cancer cell lines. (A) Relative mRNA expression of EpCAM and claudin-7 in four thyroid cancer cell lines were quantitated by RT-PCR. Expression levels were normalized by β-actin. (B) Protein expression of EpCAM and claudin-7 were analyzed by western blotting. β-actin was demonstrated as an internal control.

EpCAM is known to interact with claudin-7 and CD44v6 and plays an important role in tumor progression [16], and therefore, we tested whether both EpCAM and claudin-7 were expressed in the thyroid cancer cell lines. In the RT-PCR analysis, the expression levels of both EpCAM and caludin-7 in FRO and ACT-1 cells were significantly higher than those in TPC-1 and FTC-133 cells (Figure 3A). In the western blot analysis, the expression of EpCAM was remarkably higher in ACT-1 and FRO, than in TPC-1 and FTC-133 (Figure 3B). Thus, a significantly higher expression of EpCAM and claudin-7 in the anaplastic cancer cell lines was confirmed at both the mRNA and protein levels.

### ALDH1 enzymatic activity and mRNA expression in thyroid cancer cell lines

**Figure 4:**
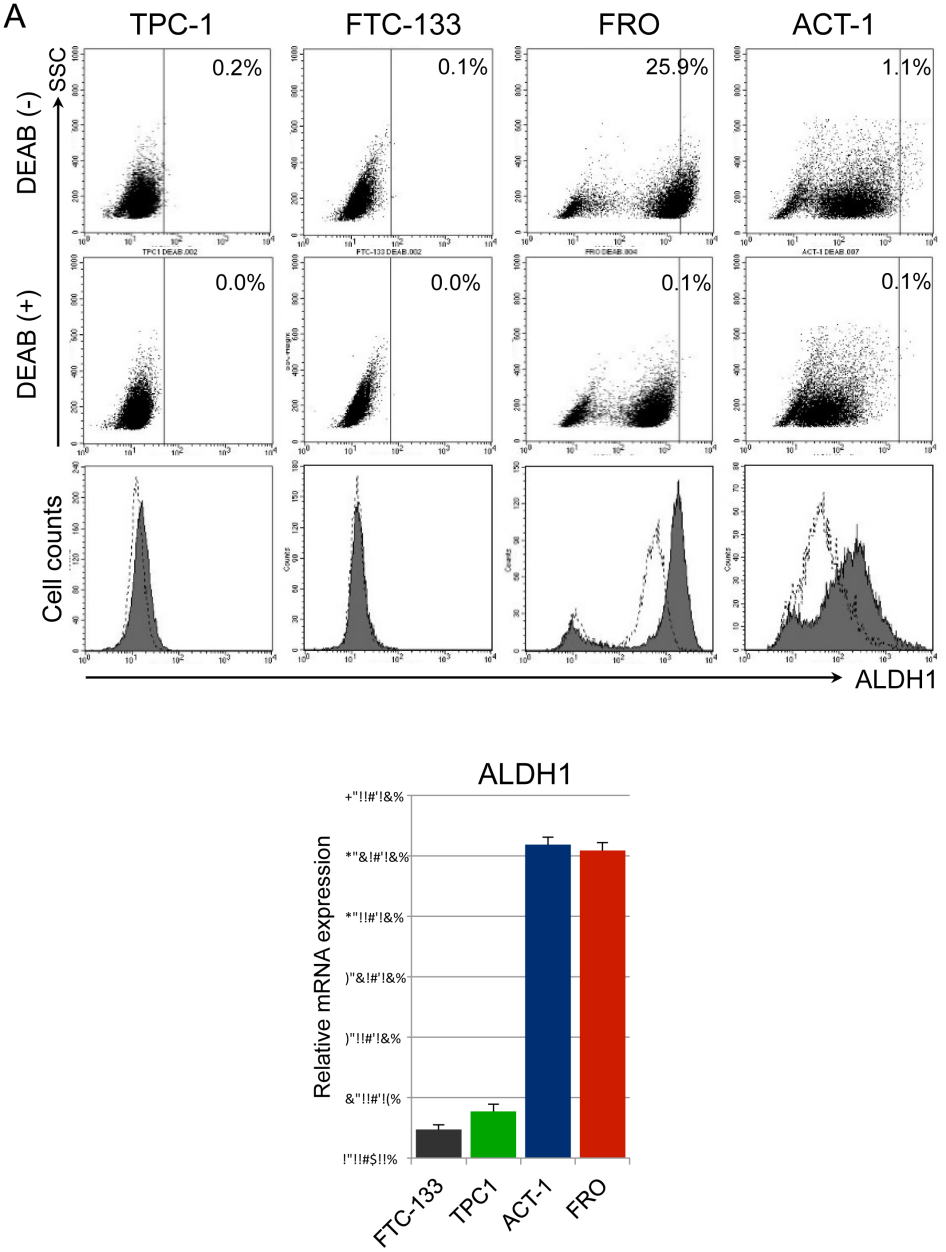
ALDH1 enzymatic activity and mRNA expression in thyroid cancer cell lines. (A) ALDH1 enzymatic activities in four thyroid cancer cell lines were detected using the ALDEFLUOR assay. DEAB was used to inhibit the ALDEFLUOR reagent, providing a negative control. Filled histograms represent the distribution of ALDH1 enzymatic activities in each cell line in the absence of DEAB, open histograms show those in the presence of DEAB. (B) Relative mRNA expressions of ALDH1 in four thyroid cancer cell lines were quantitated by RT-PCR. Expression levels were normalized by β-actin.

As CD44s has a property as a marker of tumor-initiating cells in several solid malignancies [22], [26]–[29], we tested whether or not there is an association between the expression of CD44s and human aldehyde dehydrogenase 1 (ALDH1) enzymatic activity, which is another marker of tumor-initiating cells [39], [40], in thyroid cancer cell lines. Firstly, we analyzed the ALDH1 enzymatic activity in the thyroid cancer cell lines using the Aldefluor assay. No ALDH1 enzymatic activity was detected in TPC-1 and FTC-133. However, a bimodal distribution of ALDH1 enzymatic activity was observed in both FRO and ACT-1 cells, and a part of cells showed ALDH1 enzymatic activity in both anaplastic thyroid cancer cell lines (Figure 4A). Thus, the anaplastic thyroid cancer cell lines showed a higher ALDH1 activity than the differentiated thyroid cancer cell lines.

By the ALDEFLUOR assay, the ALDH1 enzymatic activity was higher in the FRO and ACT-1 than the TPC-1 and FTC-133. We examined ALDH1 mRNA expression levels in thyroid cancer cell lines by RT-PCR (Figure 4B). By RT-PCR analysis, the expression of ALDH1 was significantly higher in FRO and ACT-1 cells than in TPC-1 and FTC-133 cells. The data indicate that the anaplastic thyroid cancer cell lines showed increased expression of ALDH1 than the differentiated thyroid cancer cell lines.

### Comparison of expression of CD44s and ALDH1 activity in thyroid cancer cell lines

**Figure 5:**
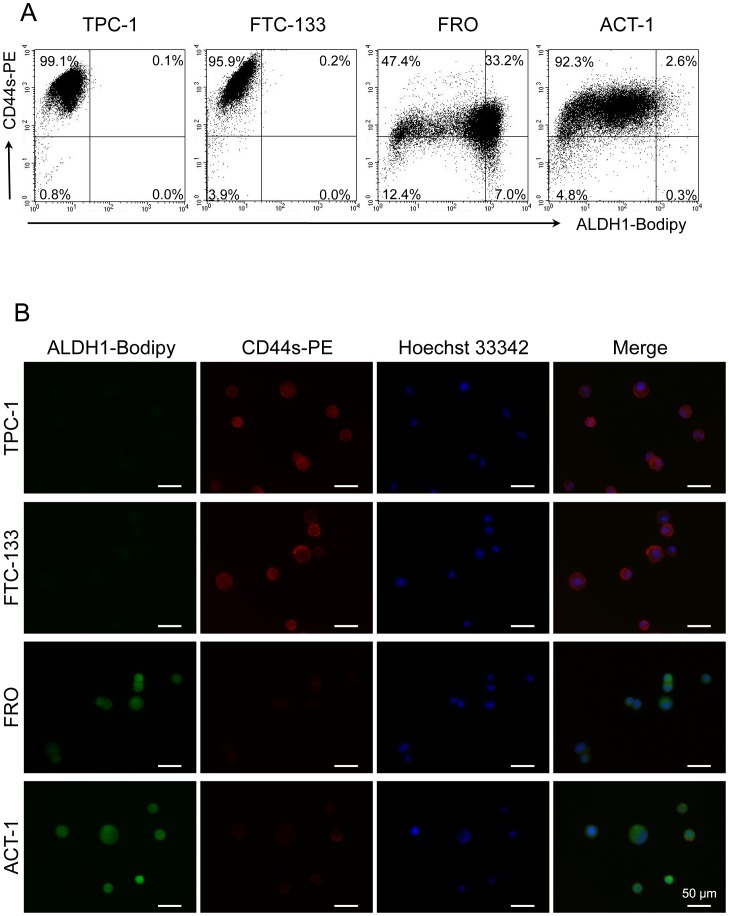
ALDH1 enzymatic activity and CD44s expression in thyroid cancer cell lines. (A) Four thyroid cancer cell lines were tested for expression of CD44s and ALDH1 enzymatic activity by flow cytometry. The number indicated the percentage of the cells in each subset. (B) Immunofluorescence finding of CD44s and ALDH1 in indicated cell lines. Fluorescence of PE-conjugated CD44s (red) and PE-conjugated ALDH1-Bodipy (green) were examined under a fluorescence microscope. Nuclei were stained blue with Hoechst 33342. Scale bar = 50 µm.

Following the experiments demonstrated above, flow cytometry analysis of ALDH1 and CD44s was conducted to analyze the association of ALDH1 activity and the expression of CD44s in the four thyroid cancer cell lines (Figure 5A, B). In TPC-1 and FTC133 cells, most of the cells expressed a high level of CD44s and low level of ALDH1 activity. In contrast, a majority of cells in FRO showed a low level of CD44s expression and a high level of ALDH1 activity (Figure 5A). ALDH1 activity was detected mostly in the cytoplasm of the cells. Furthermore, a few CD44s positive FRO cells did not show ALDH1 activity under a fluorescence microscope (Figure 5B). In ACT-1 cells, most cells expressed lower levels of CD44s than the TPC-1 or FTC-133 cells. However, strong ALDH1 activity was detected in the CD44s negative ACT-1 cells. Thus, an inverse association was observed between the expression of CD44s and ALDH1 enzymatic activity in these thyroid cancer cell lines, and the anaplastic thyroid cancer cell lines showed higher ALDH1 activity and lower CD44s expression than the differentiated thyroid cancer cell lines.

### Association of EpCAM expression and ALDH1 activity in thyroid cancer cell lines

**Figure 6:**
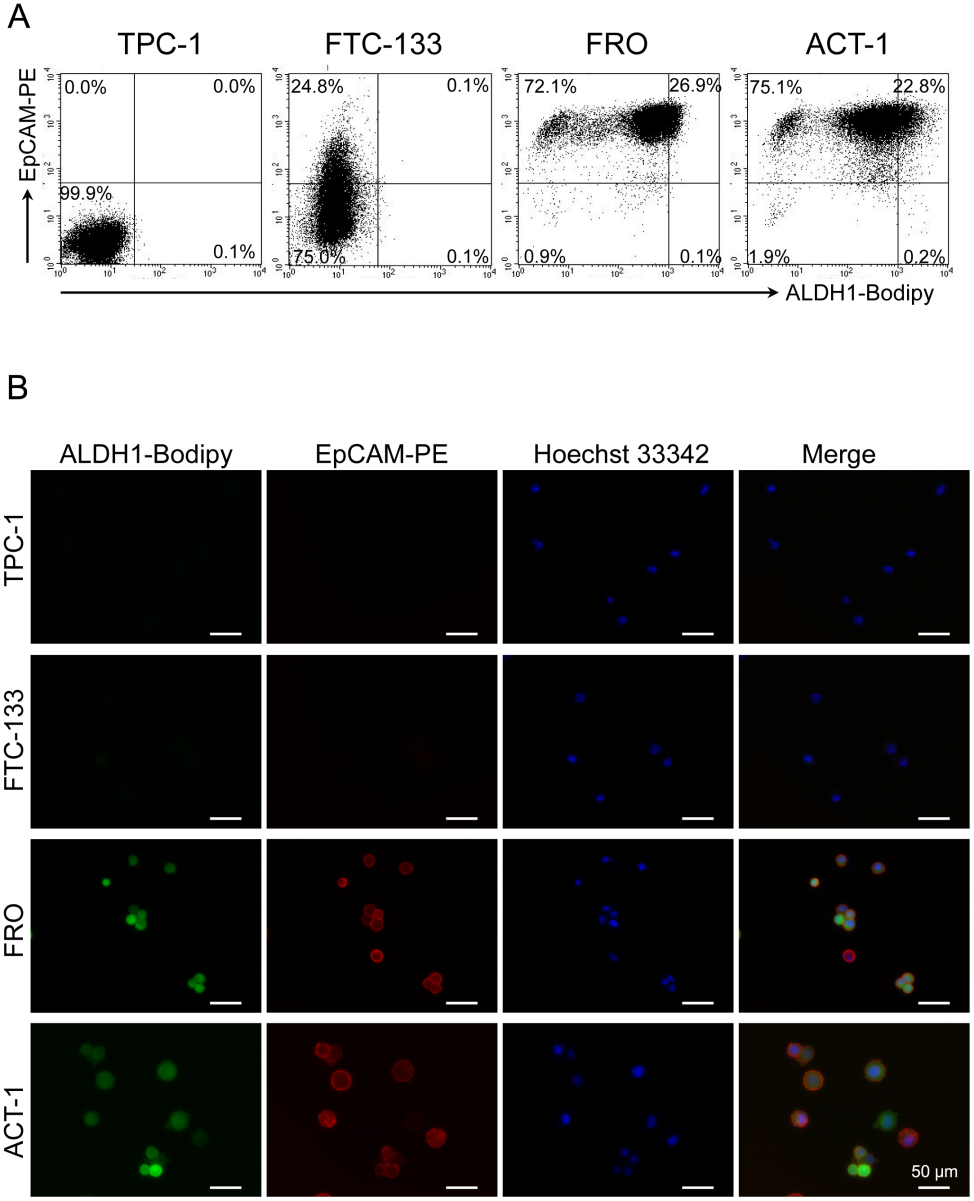
ALDH1 enzymatic activity and EpCAM expression in thyroid cancer cell lines. (A) Four thyroid cancer cell lines were tested for expression of EpCAM and ALDH1 enzymatic activity by flow cytometry. The number indicated the percentage of the cells in each subset. (B) Immunofluorescence finding of ALDH1 and EpCAM in indicated cell lines. Fluorescence of PE-conjugated EpCAM (red) and ALDH1-Bodipy (green) were examined under a fluorescence microscope. Nuclei were stained blue with Hoechst 33342. Scale bar = 50 µm.

As the expression of CD44s was inversely associated with both the expression of EpCAM and ALDH1 enzymatic activity, we analyzed whether or not there was an association between the expression of EpCAM and ALDH1 enzymatic activity (Figure 6A and B). Neither ALDH1 activity nor EpCAM expression was detectable in TPC-1 cells. In FTC-133 cells, ALDH1 activity was not detected, but a small number of cells expressed very low levels of EpCAM. In FRO and ACT-1 cells, most of the cells showed strong EpCAM expression, and the cells positive for EpCAM demonstrated moderate to high ALDH1 activity as well (Figure 6B). These results indicate that there is a positive association between the ALDH1 activity and expression of EpCAM in these thyroid cancer cell lines.

### Comparison of EpCAM expressions in anaplastic and differentiated thyroid cancers in clinical specimens

**Figure 7:**
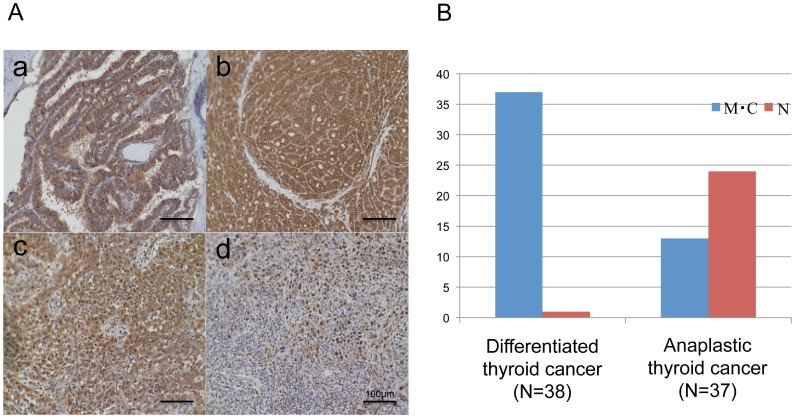
Immunohistochemical analysis of expression of EpCAM in clinical thyroid cancers. (A) Representative findings of immunohistochemical analysis of EpCAM in clinical thyroid cancer specimens; *a*, membranous and cytoplasmic staining in papillary thyroid cancer; *b*, membranous and cytoplasmic staining in follicular thyroid cancer; *c*, cytoplasmic and nuclear staining in anaplastic thyroid cancer; *d*, nuclear staining in anaplastic thyroid cancer. Scale bar = 100 µm. (B) Localization of EpCAM in the differentiated thyroid cancers and the anaplastic thyroid cancers in clinical specimens. Blue bars indicate the number of tumors showed membranous (M) or cytoplasmic (C) expression of EpCAM, and red bars indicate the number of tumors showed nuclear (N) expression.

Because higher EpCAM expression was observed in FRO and ACT-1 *in vitro* analyses, the expression of EpCAM was examined by immunohistochemistry in clinical thyroid cancer specimens (Figure 7A and B). The active form of EpCAM is known to localize to the nucleus [24], [41], and therefore, localization of EpCAM was evaluated in 38 differentiated thyroid cancers and 37 anaplastic thyroid cancers. In the differentiated thyroid cancers, membranous or cytoplasmic expression of EpCAM was detected in 37 of 38 cases, and nuclear expression of EpCAM was detected in only one case (2.6%). In contrast, nuclear expression of EpCAM was detected in 24 of 37 (64.9%) anaplastic thyroid cancers. The nuclear expression of EpCAM was significantly more frequent in the anaplastic thyroid cancers compared to the differentiated thyroid cancers. These data demonstrate that active EpCAM is present in the clinical anaplastic thyroid cancers.

### Comparison of CD44s and CD44v6 expression in anaplastic and differentiated thyroid cancers in clinical specimens

**Figure 8:**
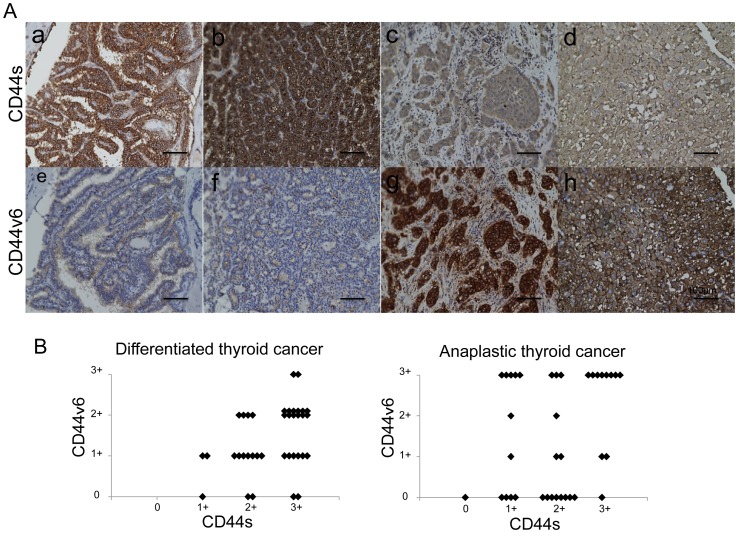
Immunohistochemical analysis of expression of CD44s and CD44v6 in clinical thyroid cancers. (A) Representative findings of immunohistochemical analysis of CD44s (*a-d*) and CD44v6 (*e-h*) in clinical thyroid cancer specimens. CD44s (*upper panel*); *a*, strong staining (3+) in papillary thyroid cancer; *b*, strong staining (3+) in follicular thyroid cancer; *c*, weak staining (1+) in anaplastic thyroid cancer, *d*, weak staining (1+) in anaplastic thyroid cancer. CD44v6 (*lower panel*); *e*, weak staining (1+) in papillary thyroid cancer; *f*, negative staining (0) in follicular thyroid cancer; *g*, strong staining (3+) in anaplastic thyroid cancer, *h*, strong staining (3+) in anaplastic thyroid cancer. Scale bar = 100 µm. (B) Association of CD44s and CD44v6 expressions in the differentiated thyroid cancers and the anaplastic thyroid cancers. The expressions of CD44s and CD44v6 of each tumor were classified into four categories according to the intensity of staining as described in the Materials and methods, and the scores are plotted in the graph.

Because reduced CD44s expression along with increased CD44v6 expression was detected in two anaplastic thyroid cancer cell lines, the expression of CD44s and CD44v6 were evaluated by immunohistochemistry in clinical specimens of thyroid cancers (Figure 8A and B). The intensity of staining was classified into four levels for CD44s and CD44v6, respectively. Anaplastic thyroid cancers showed a tendency to express lower levels of CD44s and higher levels of CD44v6 compared with the differentiated thyroid cancers. Moreover, high levels of CD44v6 and low levels of CD44s were completely not observed in differentiated thyroid cancers. In the anaplastic thyroid cancers, 16 of 37 tumors (43.2%) showed strong staining for CD44v6, while only 2 of 38 tumors (5.3%) showed strong CD44v6 expression in the differentiated carcinomas. Thus, higher expression of CD44v6 was more frequently detected in anaplastic thyroid cancers in contrast to the predominance of higher CD44s in the differentiated thyroid cancers.

### Comparison of localization of EpCAM and CD44 expression in clinical anaplastic thyroid cancers

Because an inverse association between the expression of CD44s and EpCAM along with high expression of CD44v6 were detected in two anaplastic thyroid cancer cell lines, the localization of EpCAM was compared with the expression of CD44s and CD44v6 by immunohistochemistry in 37 clinical specimens of anaplastic thyroid cancers (Table 2). The expression of CD44s was detected in 36 of 37 anaplastic thyroid cancers regardless of the localization of EpCAM. On the other hand, the expression of CD44v6 was more frequently detected in the anaplastic cancers in which the nuclear expression of EpCAM was detected, although a significant correlation was not detected. The data implies the association of EpCAM and CD44v6 in the clinical anaplastic thyroid cancers.

**Table 2:** Comparison of localization of EpCAM and CD44 expression in clinical anaplastic thyroid cancers (N = 37).

		Localization of EpCAM		*p*
		Membrane or cytoplasm	Nucleus	
CD44s	Negative	1	0	NS
	Positive	12	24	
CD44v6	Negative	6	8	NS
	Positive	7	16	

## Discussion

In the present study, we demonstrated that EpCAM, together with CD44 and claudin-7, is associated with the phenotype of anaplastic thyroid cancer both in the established cell lines and clinical specimens. The promotion of carcinogenesis and metastasis by carcinoma-specific complexes, comprised of EpCAM with CD44 variants and claudin-7, has been demonstrated in several malignancies [11]. In colorectal cancers, Kuhn et al. have reported that the EpCAM-claudin-7 complex is frequently observed in highly metastatic tumors, and the overexpression of EpCAM is associated with poor prognosis [16]. Although several studies demonstrated the association of aggressiveness of thyroid cancer and localization of EpCAM, the involvement of EpCAM in carcinoma-specific complexes with variant isoforms of CD44 and claudin-7 has not been studied in thyroid cancer. This is the first report that demonstrates the association of carcinoma-specific complexes comprised of EpCAM with the variant isoforms of CD44 and claudin-7 in thyroid cancer including anaplastic thyroid cancer.

Recent studies have demonstrated that nuclear localization of EpCAM could be a useful biomarker for aggressive thyroid cancer. Ralphan et al. demonstrated that the low-grade papillary thyroid cancers showed membranous expression of EpCAM and no detectable nuclear EpCAM. On the other hand, loss of membranous expression and increased nuclear accumulation of EpCAM is observed in the anaplastic thyroid carcinomas in the immunohistochemical analysis of clinical specimens [34]. Furthermore, He et al. demonstrated the potential of nuclear accumulation of the intracellular domain of EpCAM as a useful marker to distinguish aggressive papillary thyroid carcinoma from non-aggressive types [35]. In addition, the same group reported that nuclear and cytoplasmic intracellular domain of EpCAM and loss of membranous extracellular domain correlate with metastasis in papillary thyroid microcarcinoma [42]. In our present study, the expression of EpCAM was undetectable in FTC-133 and TPC-1 by immunocytochemistry. In contrast, immunocytochemistry analysis demonstrated that FRO and ACT-1 showed higher expression of EpCAM. Moreover, the expressions of EpCAM as analyzed by RT-PCR and western blot analysis showed results consistent with the immunocytochemistry. Our data suggest that expression of EpCAM may be upregulated in anaplastic cancer cells. In the immunohistochemical analysis of clinical specimens of thyroid cancer, the expression of EpCAM was detected on the membrane or in the cytoplasm in the differentiated thyroid cancers except one case; however, nuclear expression of EpCAM was significantly detected more frequently in anaplastic cancers compared with differentiated thyroid cancers, which is consistent with the report by Ralhan et al. Our data indicate that both expression levels and localization of EpCAM may be altered during anaplastic transformation from the differentiated thyroid carcinomas.

CD44v4–v7 are thought to be carcinoma-associated variants of CD44, which is a surface molecule that promotes tumor metastasis. Claudin-7 is a protein required for the formation of tight junctions. EpCAM has been shown to directly interact with CD44v4–v7 but not with CD44s and claudin-7 [32], [33]. In our study, flow cytometry analysis revealed an inverse association between the expression of EpCAM and CD44s was observed in thyroid cancer cell lines. In addition, double immunofluorescent staining of EpCAM and CD44s demonstrated that EpCAM was not expressed in the cells strongly positive for CD44s, but was expressed in the cells weakly positive or negative for CD44s in the thyroid cancer cell lines. The expression of CD44v6 was higher in the anaplastic thyroid cancer cell lines, which showed a lower level of CD44s compared with the differentiated thyroid cancer cell lines. Although we analyzed only CD44v6 among the carcinoma-associated variants of CD44, our data suggested the interaction between EpCAM and the other carcinoma-associated variants of CD44 in thyroid cancer as well. With regard to claudin-7, its expression was significantly higher in the anaplastic thyroid cancer cell lines, which showed a higher EpCAM expression as well. Thus, a positive association between EpCAM and claudin-7 was observed in our study, which is consistent with the direct interaction between EpCAM and claudin-7 demonstrated in the previous studies in colon cancer [16]. Although direct interaction of EpCAM, CD44 variants, and claudin-7 was not examined in our study, the results suggest that complexes of EpCAM with CD44 variants and claudin-7 may be associated with the aggressive phenotype of anaplastic thyroid cancer.

Human aldehyde dehydrogenase 1 (ALDH1) is considered as to be a marker for tumor initiating cells of several cancers such as breast, lung, pancreas, and colon. ALDH1 is believed to directly or indirectly regulate several cellular processes such as differentiation, proliferation, morphoregulation, and development, and is frequently over-expressed in tumor initiating cells. ALDH1 enzymes confer cytoprotective effects and resistance to some chemotherapeutic drugs such as cyclophosphamide [39], [40]. Thus, ALDH1 is considered one marker of tumor initiating cells. As anaplastic thyroid carcinoma is highly resistant to cytotoxic chemotherapeutic agents and effective treatment has not been established till date, these findings imply that some part of the aggressive phenotype of anaplastic thyroid cancer may attribute to the expression of ALDH1. In the present study, both enzymatic activity and mRNA expression of ALDH1 were clearly higher in the anaplastic thyroid cancer cell lines than in the differentiated thyroid cancer cell lines. Interestingly, double immunofluorescence staining assay demonstrated that ALDH1 was expressed mostly in the EpCAM positive cells in the anaplastic thyroid cancer cell lines. On the contrary, in the differentiated thyroid cancer cell lines, which were mostly negative for EpCAM, the expression of ALDH1 was rarely detected. Thus, a positive association between the expression of ALDH1 and EpCAM was observed in the thyroid cancer cell lines. In colorectal cancer emerging from chronic ulcerative colitis, a subset of cells with an ALDH1+/EpCAM+ phenotype has been described to be the cells of origin in the transition from a chronic ulcerative colitis to an overt colorectal cancer [43]. However, direct interaction of ALDH1 and EpCAM has not been fully evaluated. Our data, together with the previous report, suggest that these molecules may contribute to the development of an aggressive phenotype of malignant tumors including anaplastic thyroid carcinoma.

Double immunofluorescence staining demonstrated that most of the cells in the differentiated thyroid cancer cell lines were CD44s positive and ALDH1 negative. On the other hand, most of the cells in the anaplastic thyroid cancer cell lines were ALDH1 positive but weak or negative for the expression of CD44s. Thus, an inverse association between the expression of CD44s and that of ALDH1 was observed in the thyroid cell lines examined. Although both ALDH1 and CD44s are considered markers for tumor initiating cells, direct interaction between ALDH1 and CD44s has been rarely reported. Li et al. demonstrated that overexpression of Twist, which is involved in the process of epithelial-mesenchymal transition, leads to an upregulation of ALDH1 and CD44, and to the activation of the Akt pathway and β-catenin in HeLa and MCF7 cells [44]. In their experiments, a positive association was observed between ALDH1 and CD44. However, as the expressions of variant isoforms of CD44 were not evaluated in their experiments and the origins of the cell lines were different from those used in our study, the discrepancy should be interpreted with care. An inverse association between the expression of EpCAM and CD44s along with the co-expression of EpCAM and ALDH1 were observed in the cell lines examined in the present study, and therefore, the results may reflect the feature of a subset of cells positive for EpCAM as a primary trait regulator in thyroid cancer.

In conclusion, our study suggests the possibility that EpCAM, together with CD44v6 and claudin-7 as well as ALDH1, may be involved in the development of the aggressive phenotype of anaplastic thyroid carcinoma. However, further research is required to elucidate the precise mechanism of the transition of indolent differentiated thyroid carcinoma to virulent anaplastic thyroid carcinoma.
